# *A Priori* and *a Posteriori* Dietary Patterns during Pregnancy and Gestational Weight Gain: The Generation R Study

**DOI:** 10.3390/nu7115476

**Published:** 2015-11-12

**Authors:** Myrte J. Tielemans, Nicole S. Erler, Elisabeth T. M. Leermakers, Marion van den Broek, Vincent W. V. Jaddoe, Eric A. P. Steegers, Jessica C. Kiefte-de Jong, Oscar H. Franco

**Affiliations:** 1 Department of Epidemiology, Erasmus MC, University Medical Center Rotterdam, P.O. Box 2040, 3000 CA Rotterdam, The Netherlands; n.erler@erasmusmc.nl (N.S.E.); e.leermakers@erasmusmc.nl (E.T.M.L.); marion.vdbroek90@gmail.com (M.V.D.B.); v.jaddoe@erasmusmc.nl (V.W.V.J.); j.c.kiefte-dejong@erasmusmc.nl (J.C.K.-D.J.); o.franco@erasmusmc.nl (O.H.F.); 2 The Generation R Study Group, Erasmus MC, University Medical Center Rotterdam, P.O. Box 2040, 3000 CA Rotterdam, The Netherlands; 3 Department of Biostatistics, Erasmus MC, University Medical Center Rotterdam, P.O. Box 2040, 3000 CA Rotterdam, The Netherlands; 4 Department of Pediatrics, Erasmus MC, University Medical Center Rotterdam, P.O. Box 2040, 3000 CA Rotterdam, The Netherlands; 5 Department of Obstetrics and Gynecology, Erasmus MC, University Medical Center Rotterdam, P.O. Box 2040, 3000 CA Rotterdam, The Netherlands; e.a.p.steegers@erasmusmc.nl; 6 Department of Global Public Health, Leiden University College the Hague, P.O. Box 13228, 2501 EE the Hague, The Netherlands

**Keywords:** pregnancy, gestational weight gain, dietary pattern, maternal diet, cohort

## Abstract

Abnormal gestational weight gain (GWG) is associated with adverse pregnancy outcomes. We examined whether dietary patterns are associated with GWG. Participants included 3374 pregnant women from a population-based cohort in the Netherlands. Dietary intake during pregnancy was assessed with food-frequency questionnaires. Three *a posteriori*-derived dietary patterns were identified using principal component analysis: a “Vegetable, oil and fish”, a “Nuts, high-fiber cereals and soy”, and a “Margarine, sugar and snacks” pattern. The *a priori*-defined dietary pattern was based on national dietary recommendations. Weight was repeatedly measured around 13, 20 and 30 weeks of pregnancy; pre-pregnancy and maximum weight were self-reported. Normal weight women with high adherence to the “Vegetable, oil and fish” pattern had higher early-pregnancy GWG than those with low adherence (43 g/week (95% CI 16; 69) for highest *vs.* lowest quartile (Q)). Adherence to the “Margarine, sugar and snacks” pattern was associated with a higher prevalence of excessive GWG (OR 1.45 (95% CI 1.06; 1.99) Q4 *vs.* Q1). Normal weight women with higher scores on the “Nuts, high-fiber cereals and soy” pattern had more moderate GWG than women with lower scores (−0.01 (95% CI −0.02; −0.00) per SD). The *a priori*-defined pattern was not associated with GWG. To conclude, specific dietary patterns may play a role in early pregnancy but are not consistently associated with GWG.

## 1. Introduction

Abnormal maternal weight gain during pregnancy (*i.e.*, too little or too much) has been associated with unfavorable pregnancy outcomes in both mother and child. Insufficient gestational weight gain (GWG) is associated with both preterm birth and low birthweight [1], and excessive GWG increases the risk of giving birth to large-for-gestational-age infants [2]. Excessive GWG is also associated with maternal pregnancy complications, including hypertensive disorders [3,4] and gestational diabetes [5], which can increase the risk of the mother developing cardiometabolic diseases after pregnancy [6,7].

Energy intake during pregnancy is associated with GWG [4,8], but literature is scarce on whether GWG could be influenced by dietary composition. Some studies have examined the influence of food groups on GWG [9,10,11]. These studies found no association of fruit or vegetable intake with GWG [9,11] but unhealthier foods (e.g., sweets and processed foods) were associated with higher prevalence of excessive GWG [9,10,11]. Weight gain during pregnancy involves both maternal components (e.g., blood volume increase, fat accretion) and fetal components (e.g., weight of the fetus, amniotic fluid) [12]. Therefore, the effect of diet on weight gain may differ between pregnant and non-pregnant women.

Assessing overall diet in relation to GWG has several advantages over studying individual foods or nutrients. First, the intakes of different nutrients are often highly correlated, which complicates the assessment of individual nutrients [13]. Second, possible associations between nutrient intake and GWG might be affected by biological interactions between nutrients [13]. For these reasons, evaluating diet using a dietary pattern approach may improve our understanding of which dietary pattern is most beneficial during pregnancy. Also, this approach can facilitate future food-based dietary guidelines [14].

Only a few studies have focused on the relationship between dietary patterns and GWG [15,16,17,18]. However, no study evaluated dietary patterns and longitudinal development of weight during pregnancy. We hypothesized that specific dietary patterns may influence the development of maternal weight during pregnancy. In addition, dietary patterns are likely to differ between countries and populations [13], so it is important to identify country-specific dietary patterns that may be associated with GWG.

Hence, the purpose of our study was to determine whether *a posteriori*-derived and *a priori*-defined dietary patterns are associated with GWG during different phases in pregnancy, adequacy of GWG and weight development during pregnancy in Dutch women participating in a population-based cohort.

## 2. Experimental Section

### 2.1. Study Design

This study was embedded in the Generation R Study, a population-based prospective cohort from fetal life onwards in Rotterdam (The Netherlands). Details of this study have been described previously [19]. Briefly, pregnant women with an expected delivery date between April 2002 and January 2006, living in the urban area around Rotterdam were approached to participate. All participants provided written informed consent. The study was conducted according to the World Medical Association Declaration of Helsinki and was approved by the Medical Ethics Committee, Erasmus Medical Center Rotterdam (The Netherlands, MEC 198.782.2001.31).

### 2.2. Population of Analysis

**Figure 1:**
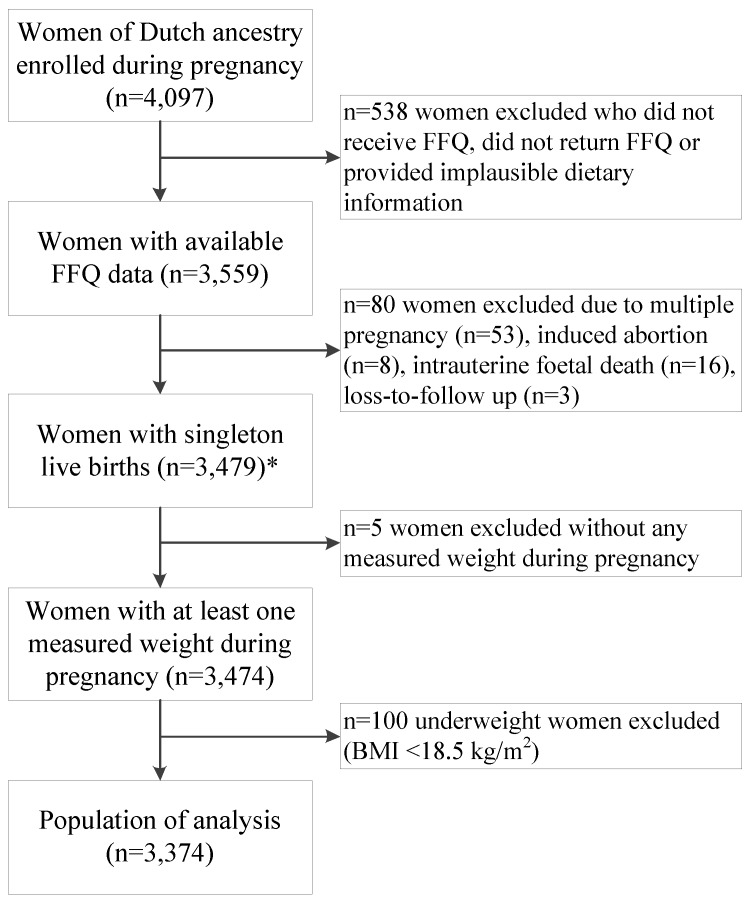
Flow chart of the study population: the Generation R Study (2002–2006). * Population in which the *a posteriori*-derived dietary patterns were determined. Abbreviations: BMI: body mass index; FFQ: food-frequency questionnaire.

For the current analysis, we included women of Dutch ancestry who entered the Generation R Study during pregnancy (*n* = 4097). We did not include women of non-Dutch ancestry because the dietary assessment method that we used was designed to evaluate a Dutch diet. We excluded women with missing dietary information (*n* = 538) and restricted our analysis to women with singleton live births (*n* = 3479). We excluded 5 women whose weight was not measured during pregnancy. Finally, we excluded women who were underweight before pregnancy (body mass index (BMI) < 18.5 kg/m^2^; *n* = 100), leaving 3374 women for the current analysis (Figure 1).

### 2.3. Dietary Assessment

Dietary intake in early pregnancy was assessed at enrolment (median 13.4 weeks of gestation (Inter Quartile Range (IQR) 12.2–15.5)) using a 293-item semi-quantitative food-frequency questionnaire (FFQ) that covered dietary intake over the previous three months. The FFQ contained questions regarding foods that are frequently consumed in a traditionally Dutch diet, their consumption frequency, portion size [20], preparation methods, and additions to foods. The average daily intake of energy and nutrients was calculated using the Dutch food-composition table (2006) [21]. The FFQ was designed for and validated in an elderly population [22], and has additionally been validated against three 24-h dietary recalls in 71 Dutch pregnant women who visited a midwifery in Rotterdam. The intra-class correlation coefficients for energy-adjusted macronutrients ranged between 0.48 and 0.68.

#### 2.3.1. *A Posteriori*-Derived Dietary Patterns

We used principal component analysis (PCA) with Varimax rotation to identify *a posteriori*-derived dietary patterns [13,23]. Our dietary patterns have been described in detail previously [24]. Briefly, the 293 individual food items from the FFQ were aggregated into 23 food groups (Table S1). Subsequently, we extracted those factors (*i.e.*, dietary patterns) of the PCA that had an Eigenvalue of ≥1.5 [25]. The factor loadings, which described how strong the association between the food groups and each of the extracted patterns is, are presented in Table 1. Finally, we determined factor scores (*i.e.*, adherence scores) for each participant and each pattern, by calculating the individual sum of the intake of the food groups, weighted with their factor loadings and standardizing those weighted sums to have mean zero and standard deviation one (standard deviation score). A higher factor score indicated that a woman’s diet was closer to that dietary pattern.

Three *a posteriori*-derived dietary patterns were identified, namely a “Vegetable, oil and fish” pattern, a “Nuts, high-fiber cereals and soy” pattern and a “Margarine, sugar and snacks” pattern, together explaining 25.8% of the variance in maternal dietary intake (Table 1).

**Table 1:** Factor loadings food groups in *a posteriori*-derived dietary patterns ^1^.

Food Group	“Vegetable, Oil and Fish” Dietary Pattern	“Nuts, High-Fiber Cereals and Soy” Dietary Pattern	“Margarine, Sugar and Snacks” Dietary Pattern
Potatoes and other tubers	0.05	**−0.53**	**0.21**
Vegetables	0.78 *	0.17	−0.03
Fruits	0.13	**0.37**	0.02
Dairy products—high fat	**0.26**	**−0.26**	**0.29**
Dairy products—low fat	−0.15	**0.29**	0.16
Cereals—high fiber	**0.24**	0.43 *	**0.36**
Cereals—low fiber	**0.23**	−0.16	**0.25**
Meat and meat products	0.08	**−0.54**	**0.33**
Fish and shellfish	0.45 *	**0.24**	−0.11
Eggs and egg products	**0.27**	0.05	0.19
Vegetable oils	0.74 *	0.08	−0.12
Margarine and butter	−0.06	−0.03	0.61 *
Sugar and confectionary and cakes	−0.11	0.13	0.56 *
Snacks	0.05	0.08	0.40 *
Coffee and tea	**0.28**	**0.34**	0.10
Sugar-containing beverages	−0.14	**−0.28**	**0.29**
Light soft drinks	0.13	**0.28**	0.02
Alcoholic beverages	**0.35**	−0.00	−0.04
Condiments and sauces	0.05	−0.09	**0.39**
Soups and bouillon	0.19	−0.02	0.15
Nuts, seeds and olives	0.03	0.64 *	**0.30**
Soy products	0.01	0.39 *	−0.10
Legumes	**0.44**	−0.02	0.07

^1^ Reprinted with permission from van den Broek *et al.* [24]. The food groups that are considered to have a strong association with a dietary pattern (factor loading ≥0.2 or ≤−0.2) are shown in bold. The three factor loadings with the highest positive factor loading are used to name the dietary pattern and are presented with an asterisk (*). The three dietary patterns together explained 25.8% of the total variance in maternal dietary intake.

#### 2.3.2. *A Priori*-Defined Dietary Pattern

The *a priori*-defined dietary pattern was based on the Dutch Healthy Diet Index [26]. This index was developed to measure adherence to the Dutch guidelines for a healthy diet [27] and consisted of ten components: physical activity, vegetable, fruit, dietary fiber, fish, saturated fatty acids, trans-fatty acids, consumption of acidic drinks and foods, sodium, and alcohol. We omitted the components physical activity, trans-fatty acids, and the consumption of acidic drinks and foods because this information had not been collected. Furthermore, we did not include the alcohol component because alcohol abstinence is recommended during pregnancy. The score of each component ranged between 0 and 10 points, resulting in a total score ranging from 0 to 60 points (Table S2). A higher score on the Dutch Healthy Diet Index corresponds with a higher adherence to the 2006 Dutch healthy diet guidelines and thus reflects a healthier diet. Finally, to facilitate comparison between all dietary patterns, we standardized the “Dutch Healthy Diet Index” pattern to a standard deviation score.

### 2.4. Maternal Weight Gain

Information on pre-pregnancy weight was collected at enrollment using a questionnaire and was used to calculate a pre-pregnancy BMI (kg/m^2^). Women visited our research center three times at median (IQR) gestational ages of 12.9 (12.1–14.4) weeks (*first visit*), 20.4 (19.9–21.1) weeks (*second visit*), and 30.2 (29.9–30.8) weeks (*third visit*). During each visit, maternal height and weight were measured without shoes and heavy clothing. Six weeks after childbirth, women were asked to report their highest weight during pregnancy using a questionnaire, which we used as maximum weight in pregnancy.

Pre-pregnancy weight was highly correlated with weight measured during *the first visit* (*R* = 0.96, *p*-value < 0.001, *n* = 2425), and there was no indication for systematic measurement error (Figure S1). Also, a high correlation was found between weight during *the third visit* and maximum weight in pregnancy (*R* = 0.89, *p*-value < 0.001, *n* = 2177) without an indication for systematic measurement error (Figure S1). To evaluate long-term maternal weight gain, we measured maternal weight at our research center six years after childbirth.

#### 2.4.1. Gestational Weight Gain during Different Phases in Pregnancy

GWG in different phases of pregnancy was calculated for three consecutive periods, namely early-pregnancy GWG (calculated as weight at *the first visit* minus pre-pregnancy weight, divided by follow-up duration (g/week), *n* = 2425), mid-pregnancy GWG (calculated as weight at *the second visit* minus weight *the first visit*, divided by follow-up duration (g/week), *n* = 2748), and late-pregnancy GWG (calculated as weight at *the third visit* minus weight at *the second visit*, divided by follow-up duration (g/week), *n* = 3158). GWG until early-third trimester was calculated as weight at *the third visit* minus pre-pregnancy weight, divided by follow up duration (g/week, *n* = 2815).

#### 2.4.2. Adequacy of Gestational Weight Gain

Women’s total GWG (calculated as maximum weight in pregnancy minus pre-pregnancy weight, *n* = 1917) was used to classify their GWG into inadequate, adequate, or excessive GWG. Cut-off values of GWG adequacy were based on recommendations published by the US Institute of Medicine (2009) and were BMI-specific [28]. Normal weight women (BMI 18.5–24.9 kg/m^2^) were categorized as having an adequate GWG with a GWG between 11.5 and 16 kg, overweight women (BMI 25–29.9 kg/m^2^) were classified as adequate GWG with GWG between 7 and 11.5 kg, and adequate GWG for obese women (BMI ≥ 30 kg/m^2^) was between 5 and 9 kg.

### 2.5. Covariates

Several maternal sociodemographic and lifestyle characteristics were considered as potential confounders. We obtained information from prenatal questionnaires that were sent in different trimesters regarding maternal age, educational level [29], household income (≤2200 *vs.* >2200 Euro/month), parity (no child *vs.* ≥1 child), pre-pregnancy weight, pre-existing comorbidities, vomiting, smoking or alcohol consumption (both categorized as never during pregnancy, stopped when pregnancy was known, or continued throughout pregnancy), folic acid supplementation (started periconceptionally, started first 10 weeks, or no supplementation), energy intake, and stress during pregnancy (using the Global Severity Index [30]). To calculate pre-pregnancy BMI, height was measured at enrollment. Gestational age was determined based on ultrasound examination, and during *the third visit* an ultrasound was performed to estimate fetal weight. Information on fetal sex was obtained from delivery reports.

### 2.6. Statistical Analyses

We considered two sets of possible confounders in the analysis. *Model 1* was adjusted for median gestational age at follow-up and pre-pregnancy BMI. *Model 2* was further adjusted for age, educational level, household income, parity, smoking during pregnancy, alcohol consumption during pregnancy, stress during pregnancy, and fetal sex. The selection of potential confounders was based on factors found in the literature and on a change of at least 10% in effect estimate in a preliminary analysis assessing the association of dietary patterns with GWG until early-third trimester. As GWG is related to BMI [28] and the preliminary analysis showed significant interaction terms for the “Vegetable, oil and fish” pattern (*p*-value < 0.01) and the “Nuts, high-fiber cereals and soy” pattern (*p*-value = 0.01) with pre-pregnancy BMI, we stratified all analyses on the basis of weight status (normal weight (BMI < 25 kg/m^2^) and overweight (BMI ≥ 25 kg/m^2^)).

In order to adequately estimate the relationship between diet and trajectories of gestational weight in the presence of incomplete covariates, we performed a longitudinal analysis using linear mixed modelling in the Bayesian framework. This method has been described in detail previously [31]. Briefly, by modelling the joint distribution of exposure, outcome and covariates, all available information is used to impute the missing values and estimate the parameters of interest simultaneously.

In the Bayesian linear mixed model, all main effects from *Model 2*, interaction terms between the dietary pattern variables (as derived by PCA) and a linear and quadratic effect for gestational age were included in the fixed effects structure. The correlation between the weight measurements within an individual was modelled by including random effects for the intercept and slope (for gestational age) into the model. No additional correlation structure was assumed for the error terms. For this analysis, the reported parameter estimates and 95% credible intervals were obtained by taking the mean and 2.5% and 97.5% quantiles of the posterior sample of the respective parameters.

To analyze the association of the *a priori*-defined and *a posteriori*-derived dietary patterns with GWG during different phases in pregnancy, GWG until early third trimester and maximal GWG, we performed multivariable linear regression analysis. Missing covariate values were multiply imputed by randomly drawing ten values from the posterior samples of each incomplete covariate derived in the Bayesian analysis. Missing observations of gestational weight were not imputed. The reported results from the cross-sectional models were pooled over all ten completed datasets. Separate models were fitted with the dietary patterns discretized in quartiles, with the lowest quartile (quartile 1) as a reference category, as well as continuously per SD score. Quartiles were constructed separately for normal weight and overweight women; each of these analyses was done for *Model 1* and *Model 2*. To identify cases that have an influence on the regression models we calculated Cook’s distance [32].

Because GWG is a physiological process in pregnancy and resulting in weight gain in almost all women during pregnancy [28], we also evaluated the associations between dietary patterns and GWG adequacy (inadequate, adequate *vs.* excessive GWG) using multinomial regression models. We included all covariates from *Model 2* and used “adequate GWG” as a reference category.

#### Sensitivity Analyses

To test the stability of our results, we performed four sensitivity analyses in *Model 2* for the association between dietary patterns and GWG until early-third trimester. First, because energy intake may be an intermediate factor in the association of maternal diet with GWG, we further adjusted for energy intake (kcal/day). Second, we further adjusted for estimated fetal weight in the early-third trimester to evaluate whether higher GWG could be explained by greater fetal growth because we previously found that specific dietary patterns may be associated with fetal weight [33]. Third, we excluded women with pre-existing comorbidities (*n* = 182) and women with hypertensive complications in pregnancy [34] or gestational diabetes (*n* = 272) since these conditions may influence both dietary intake and GWG. Fourth, we excluded women who reported vomiting more than once per week during the three months prior to enrolment (*n* = 421), since this might alter dietary intake and GWG. Also, we explored effect modification of the association between dietary patterns and GWG with educational level and household income.

Additionally, we evaluated whether the associations of dietary patterns with GWG would markedly change when using self-reported maximum weight during pregnancy instead of measured weight at *the third visit* (*n* = 1917). Furthermore, we evaluated whether the associations found between the dietary patterns and adequacy of weekly GWG (between *the first* and *the third visit*) were similar to those with adequacy of total GWG (*n* = 2745), because some measurement error was found in the self-reported weights (Figure S1). The cut-off values of adequate weekly GWG were 0.35–0.50 kg/week for normal weight women, 0.23–0.33 kg/week of overweight women, and 0.17–0.27 kg/week for obese women [28]. In addition, we explored long-term maternal weight gain and evaluated whether this long-term weight gain differed in women with inadequate, adequate or excessive GWG using Analysis of Variance (ANOVA). Finally, we calculated the correlation between weight at *the third visit* and weight 6 years after childbirth.

All statistical analyses were performed in SPSS version 21.0 (IBM Corp., Armonk, NY, USA), R version 3.2.1 (R Foundation for Statistical Computing, Vienna, Austria) and JAGS version 3.4.0 [35].

## 3. Results

### 3.1. Study Population

Baseline characteristics for normal weight women (*n* = 2544; 75%) and overweight women (*n* = 830; 25%) are presented in Table 2. The mean score ±SD on the Dutch Healthy Diet Index was 32 ± 8 and ranged from 8 to 59. Overall, 43% of women had excessive GWG (*n* = 826); excessive GWG was found in 37% of the normal weight women (*n* = 557) and in 63% of the overweight women (*n* = 269).

We did not identify women with large influence on the effect estimates of the association between dietary patterns and GWG (all Cook’s distances were <1).

**Table 2:** Subject characteristics (*n* = 3374), the Generation R Study (2002–2006) ^1^.

Subject Characteristics	Normal Weight Women (*n* = 2544)	Overweight Women (*n* = 830)
Age (years)	31.6 ± 4.3	31.0 ± 4.4
Educational level, *n* (%)		
Low and midlow	307 (12.1)	201 (24.2)
Midhigh	1283 (50.4)	436 (52.5)
High	954 (37.5)	193 (23.3)
Household income, *n* (%)	**	
<2200 Euro/month	620 (24.4)	266 (32.1)
≥2200 Euro/month	1924 (75.6)	564 (67.9)
Parity, *n* (%)		
0	1554 (61.1)	465 (56.0)
≥1	990 (38.9)	365 (44.0)
Pre-pregnancy BMI (kg/m^2^)	21.6 (20.4–23.0)	27.7 (26.0–30.5)
Smoking during pregnancy, *n* (%)		
Never during pregnancy	1911 (75.1)	612 (73.7)
Until pregnancy was known	233 (9.2)	61 (7.3)
Continued throughout pregnancy	400 (15.7)	157 (19.0)
Alcohol consumption during pregnancy, *n* (%)		
Never during pregnancy	764 (30.0)	359 (43.2)
Until pregnancy was known	416 (16.4)	138 (16.6)
Continued throughout pregnancy	1364 (53.6)	334 (40.2)
Stress during pregnancy (score 0–4)	0.12 (0.06–0.24)	0.13 (0.06–0.26)
Energy intake (kcal/day)	2162 ± 507	2090 ± 514
Dutch Healthy Diet Index (score 0–60)	32 ± 8	30 ± 8
Fetal sex, *n* (%)		
Male	1287 (50.6)	415 (50.0)
Female	1257 (49.4)	415 (50.0)
Gestational weight gain (kg)	14.7 ± 7.3	12.9 ± 7.7
Adequacy of gestational weight gain, *n* (%)		
Inadequate	370 (24.8)	89 (20.9)
Adequate	565 (37.9)	67 (15.8)
Excessive	557 (37.3)	269 (63.3)

^1^ Values represent *n* (%) for categorical variables, and for continuous variables they represent mean ± SD or median (interquartile range). Missing data: educational level (1.3%), household income (10.3%), parity (0.2%), pre-pregnancy BMI (14.2%), smoking during pregnancy (7.4%), alcohol consumption during pregnancy (8.1%), stress during pregnancy (12.0%), gestational weight gain (43.2%), adequacy of gestational weight gain (43.2%). No missing data for maternal age, energy intake, Dutch Healthy Diet Index or fetal sex. Numbers may not add up to total due to rounding after imputation.

### 3.2. Dietary Patterns and Gestational Weight Gain in Different Phases in Pregnancy

Normal weight women in the highest quartile of the “Vegetable, oil and fish” pattern had a 43 g/week (95% CI 16; 69) greater early-pregnancy GWG than women in the lowest quartile, independent of lifestyle and sociodemographic variables. We observed no such association in overweight women (Table 3). The “Nuts, high-fiber cereals and soy” pattern was associated with a lower early-pregnancy GWG in *Model 1* in both normal weight and overweight women. However, after additional adjustment (*Model 2*) this pattern was no longer significantly associated with early-pregnancy GWG. Neither the “Margarine, sugar and snacks” pattern nor the “Dutch Healthy Diet Index” pattern was associated with early-pregnancy GWG.

**Table 3:** Association of dietary patterns with gestational weight gain in early pregnancy (*n* = 2425) ^1^.

Quartiles of the Dietary Patterns	Early-Pregnancy Weight Gain (g/Week)			
	Normal Weight Women (*n* = 1849)		Overweight Women (*n* = 576)	
	*Model 1*	*Model 2*	*Model 1*	*Model 2*
	**“Vegetable, Oil And Fish” Pattern**			
Q1 (low)	Reference	Reference	Reference	Reference
Q2	−8 (−34; 17)	−3 (−28; 23)	18 (−44; 80)	29 (−34; 91)
Q3	−14 (−40; 11)	−4 (−30; 22)	59 (−3; 121)	**77 (14; 141)**
Q4 (high)	38 (12; 63) *	43 (16; 69) *	4 (−58; 66)	31 (−37; 99)
Per SD	*p* < 0.01 *	*p* < 0.01 *	*p* = 0.63	*p* = 0.24
	**“Nuts, High-Fiber Cereals and Soy” Pattern**			
Q1 (low)	Reference	Reference	Reference	Reference
Q2	−10 (−36; 15)	5 (−21; 30)	−19 (−81; 43)	−17 (−79; 45)
Q3	**−26 (−52; −1)**	−4 (−31; 23)	−54 (−117; 10)	−44 (−109; 21)
Q4 (high)	**−31 (−57; −6)**	−10 (−37; 18)	−64 (−128; 1)	−52 (−120; 15)
Per SD	***p*** < 0.01 *	*p* = 0.22	***p* = 0.02**	*p* = 0.06
	**“Margarine, Sugar and Snacks” Pattern**			
Q1 (low)	Reference	Reference	Reference	Reference
Q2	3 (−22; 29)	2 (−23; 27)	33 (−28; 94)	28 (−32; 88)
Q3	5 (−21; 30)	−1 (−26; 24)	35 (−29; 98)	41 (−22; 103)
Q4 (high)	20 (−6; 46)	13 (−12; 39)	52 (−9; 114)	45 (−17; 106)
Per SD	*p* = 0.11	*p* = 0.36	*p* = 0.20	*p* = 0.24
	**“Dutch Healthy Diet Index” Pattern**			
Q1 (low)	Reference	Reference	Reference	Reference
Q2	0 (−25; 26)	−2 (−27; 23)	4 (−58; 65)	3 (−58; 64)
Q3	16 (−10; 42)	7 (−19; 32)	48 (−14; 110)	38 (−23; 100)
Q4 (high)	3 (−22; 29)	−14 (−40; 12)	34 (−28; 96)	11 (−54; 75)
Per SD	*p* = 0.86	*p* = 0.17	*p* = 0.32	*p* = 0.86

^1^ Results from multivariable linear regression analyses, based on imputed data. Values (regression coefficients with 95%-confidence interval) reflect the difference in early-pregnancy weight gain (g/week) for quartile 2 until 4 relative to quartile 1. *p*-Values correspond to the effect of 1SD increase in dietary pattern score. *Model 1*: adjusted for pre-pregnancy BMI and median gestational age at follow-up. *Model 2*: *Model 1* further adjusted for age, educational level, household income, parity, smoking during pregnancy, alcohol consumption during pregnancy, stress during pregnancy, and fetal sex. *p* For interaction between dietary patterns and pre-pregnancy BMI was <0.10 for the “Vegetable, oil and fish” pattern and for the other patterns >0.10. Significant results are presented in bold (*p*-value < 0.05) and results with a *p*-value < 0.0125 with an asterisk (*). Abbreviations: BMI: body mass index; Q: quartile; SD: standard deviation.

No significant associations were found for any of the dietary patterns with mid-pregnancy GWG in normal weight or overweight women (Table 4). Table 5 shows that in normal weight women, only the “Nuts, high-fiber cereals and soy” pattern was inversely associated with late-pregnancy GWG in *Model 1* (*p*-value for 1SD increase < 0.01), but these results largely attenuated after adjustment for sociodemographic and lifestyle factors (*p*-value = 0.48). In overweight women, none of the dietary patterns were significantly associated with late-pregnancy GWG.

**Table 4:** Association of dietary patterns with gestational weight gain in mid-pregnancy (*n* = 2748) ^1^.

Quartiles of the Dietary Patterns	Mid-Pregnancy Weight Gain (g/Week)			
	Normal Weight Women (*n* = 2079)		Overweight Women (*n* = 669)	
	*Model 1*	*Model 2*	*Model 1*	*Model 2*
	**“Vegetable, Oil and Fish” Pattern**			
Q1 (low)	Reference	Reference	Reference	Reference
Q2	−0 (−39; 38)	6 (−33; 45)	37 (−42; 115)	17 (−64; 97)
Q3	2 (−36; 39)	12 (−27; 51)	21 (−59; 101)	7 (−76; 90)
Q4 (high)	−13 (−52; 25)	−4 (−44; 36)	23 (−56; 103)	−19 (−105; 68)
Per SD	*p* = 0.48	*p* = 0.72	*p* = 0.36	*p* = 0.92
	**“Nuts, High-Fiber Cereals and Soy” Pattern**			
Q1 (low)	Reference	Reference	Reference	Reference
Q2	22 (−16; 60)	25 (−14; 64)	28 (−52; 107)	8 (−73; 89)
Q3	−7 (−46; 31)	−2 (−42; 39)	47 (−33; 128)	19 (−65; 102)
Q4 (high)	25 (−13; 64)	30 (−11; 70)	62 (−19; 142)	17 (−68; 103)
Per SD	*p* = 0.38	*p* = 0.32	*p* = 0.14	*p* = 0.72
	**“Margarine, Sugar and Snacks” Pattern**			
Q1 (low)	Reference	Reference	Reference	Reference
Q2	31 (−7; 68)	31 (−6; 69)	25 (−53; 102)	30 (−47; 107)
Q3	15 (−23; 53)	18 (−21; 56)	8 (−71; 87)	17 (−62; 96)
Q4 (high)	16 (−22; 54)	18 (−20; 57)	14 (−64; 92)	24 (−54; 103)
Per SD	*p* = 0.44	*p* = 0.40	*p* = 0.65	*p* = 0.48
	**“Dutch Healthy Diet Index” Pattern**			
Q1 (low)	Reference	Reference	Reference	Reference
Q2	−15 (−53; 23)	−14 (−52; 24)	−36 (−113; 41)	−31 (−109; 47)
Q3	−0 (−38; 37)	−1 (−39; 36)	−23 (−101; 54)	−4 (−81; 74)
Q4 (high)	−7 (−46; 31)	−10 (−49; 30)	−9 (−89; 70)	27 (−56; 109)
Per SD	*p* = 0.66	*p* = 0.76	*p* = 0.43	*p* = 0.88

^1^ Results from multivariable linear regression analyses, based on imputed data. Values (regression coefficients with 95%-confidence interval) reflect the difference in mid-pregnancy weight gain (g/week) for quartile 2 until 4 relative to quartile 1. *p*-Values correspond to the effect of 1SD increase in dietary pattern score. *Model 1*: adjusted for pre-pregnancy BMI and median gestational age at follow-up. *Model 2*: *Model 1* further adjusted for age, educational level, household income, parity, smoking during pregnancy, alcohol consumption during pregnancy, stress during pregnancy, and fetal sex. *p* For interaction between dietary patterns and pre-pregnancy BMI was <0.10 for the “Dutch Healthy Diet Index” pattern and for the other patterns >0.10. Abbreviations: BMI: body mass index; Q: quartile; SD: standard deviation.

**Table 5:** Association of dietary patterns with gestational weight gain in late pregnancy (*n* = 3158) ^1^.

Quartiles of the Dietary Patterns	Late-Pregnancy Weight Gain (g/week)			
	Normal Weight Women (*n* = 2384)		Overweight Women (*n* = 774)	
	*Model 1*	*Model 2*	*Model 1*	*Model 2*
	**“Vegetable, Oil and Fish” Pattern**			
Q1 (low)	Reference	Reference	Reference	Reference
Q2	−3 (−32; 26)	10 (−20; 39)	21 (−35; 78)	36 (−21; 93)
Q3	−18 (−47; 10)	−4 (−33; 26)	−3 (−60; 55)	21 (−38; 80)
Q4 (high)	−19 (−47; 10)	−0 (−31; 30)	−8 (−64; 49)	24 (−38; 86)
Per SD	*p* = 0.09	*p* = 0.54	*p* = 0.42	*p* = 0.82
	**“Nuts, High-Fiber Cereals and Soy” Pattern**			
Q1 (low)	Reference	Reference	Reference	Reference
Q2	−2 (−30; 27)	14 (−15; 44)	4 (−54; 62)	18 (−41; 77)
Q3	−16 (−45; 12)	8 (−22; 38)	−3 (−60; 55)	15 (−45; 74)
Q4 (high)	**−37 (−65; −8)**	−13 (−43; 18)	3 (−55; 61)	21 (−41; 83)
Per SD	***p*** < 0.01 *	*p* = 0.48	*p* = 0.91	*p* = 0.66
	**“Margarine, Sugar and Snacks” Pattern**			
Q1 (low)	Reference	Reference	Reference	Reference
Q2	−21 (−49; 8)	−20 (−48; 8)	−8 (−65; 49)	−7 (−63; 50)
Q3	−12 (−41; 16)	−12 (−40; 17)	7 (−49; 64)	17 (−40; 74)
Q4 (high)	−5 (−34; 24)	−6 (−35; 23)	8 (−49; 65)	10 (−48; 68)
Per SD	*p* = 0.86	*p* = 0.76	*p* = 0.64	*p* = 0.66
	**“Dutch Healthy Diet Index” Pattern**			
Q1 (low)	Reference	Reference	Reference	Reference
Q2	−14 (−43; 14)	−13 (−41;15)	46 (−10; 102)	51 (−5; 108)
Q3	−2 (−31; 27)	−10 (−39; 18)	23 (−34; 81)	25 (−33; 82)
Q4 (high)	−3 (−31; 26)	−14 (−43; 15)	33 (−24; 90)	28 (−31; 88)
Per SD	*p* = 0.61	*p* = 0.57	*p* = 0.46	*p* = 0.58

^1^ Results from multivariable linear regression analyses, based on imputed data. Values (regression coefficients with 95%-confidence interval) reflect the difference in late-pregnancy weight gain (g/week) for quartile 2 until 4 relative to quartile 1. *p*-Values correspond to the effect of 1SD increase in dietary pattern score. *Model 1*: adjusted for pre-pregnancy BMI and median gestational age at follow-up. *Model 2*: *Model 1* further adjusted for age, educational level, household income, parity, smoking during pregnancy, alcohol consumption during pregnancy, stress during pregnancy, and fetal sex. *p* For interaction between dietary patterns and pre-pregnancy BMI was >0.10 for the “Nuts, high-fiber cereals and soy” and the “Dutch Healthy Diet Index” pattern, but <0.10 for the “Vegetable, oil and fish” and the “Margarine, sugar and snacks” pattern. Significant results are presented in bold (*p*-value < 0.05) and results with a *p*-value < 0.0125 with an asterisk (*). Abbreviations: BMI: body mass index, Q: quartile, SD: standard deviation.

In line with the results from early-pregnancy GWG, normal weight women in the highest quartile of the “Vegetable, oil and fish” pattern had higher GWG by 25 g/week (95% CI 9; 42) until the early-third trimester than women in the lowest quartile (Table S3), whereas no association was found in overweight women (Table S4). The other dietary patterns were not associated with GWG until the early-third trimester.

### 3.3. Dietary Patterns and Gestational Weight Gain Adequacy

Higher adherence to the dietary patterns was not associated with the prevalence of inadequate GWG (Table 6). The “Vegetable, oil and fish”, the “Nuts, high-fiber cereals and soy” and the “Dutch Healthy Diet Index” pattern were also not associated with prevalence of excessive GWG. Yet, women with higher scores on the “Margarine, sugar and snacks” pattern had a higher prevalence of excessive GWG than women in the lowest quartile (ORs Q2: 1.40 (95% CI 1.04; 1.90), Q3: 1.37 (95% CI 1.00; 1.87), and Q4: 1.45 (95% CI 1.06; 1.99)).

**Table 6:** Association of dietary patterns with gestational weight gain adequacy (*n* = 1917) ^1^.

Quartiles of the Dietary Patterns	Inadequate GWG (*n* = 459)	Adequate GWG (*n* = 632)	Excessive GWG (*n* = 826)
	OR (95% CI)		OR (95% CI)
	**“Vegetable, Oil and Fish” Pattern**		
Q1 (low)	Reference	Reference	Reference
Q2	0.85 (0.60; 1.22)	Reference	1.08 (0.79; 1.48)
Q3	0.86 (0.60; 1.23)	Reference	1.05 (0.76; 1.46)
Q4 (high)	0.84 (0.58; 1.22)	Reference	1.06 (0.76; 1.48)
Per SD	*p* = 0.21		*p* = 0.91
	**“Nuts, High-Fiber Cereals and Soy” Pattern**		
Q1 (low)	Reference	Reference	Reference
Q2	0.77 (0.53; 1.13)	Reference	1.16 (0.82; 1.62)
Q3	0.86 (0.59; 1.25)	Reference	1.26 (0.89; 1.77)
Q4 (high)	0.85 (0.58; 1.24)	Reference	1.09 (0.77; 1.53)
Per SD	*p* = 0.76		*p* = 0.46
	**“Margarine, Sugar and Snacks” Pattern**		
Q1 (low)	Reference	Reference	Reference
Q2	0.97 (0.69; 1.36)	Reference	**1.40 (1.04; 1.90)**
Q3	0.93 (0.66; 1.32)	Reference	**1.37 (1.00; 1.87)**
Q4 (high)	0.98 (0.69; 1.40)	Reference	**1.45 (1.06; 1.99)**
Per SD	*p* = 0.73		*p* = 0.09
	**“Dutch Healthy Diet Index” Pattern**		
Q1 (low)	Reference	Reference	Reference
Q2	1.04 (0.74; 1.45)	Reference	0.92 (0.69; 1.24)
Q3	0.84 (0.59; 1.20)	Reference	0.95 (0.70; 1.27)
Q4 (high)	1.32 (0.92; 1.90)	Reference	1.11 (0.80; 1.53)
Per SD	*p* = 0.07		*p* = 0.66

^1^ Results (OR with 95%-confidence interval) from multivariable multinomial logistic regression analyses, based on imputed data. Low dietary pattern adherence (Q1) is the reference category for diet and adequate GWG is the reference category for adequacy of GWG in the multinomial regression model. *p*-Values correspond to the effect of 1SD increase in dietary pattern score. Adjusted for pre-pregnancy BMI, age, educational level, household income, parity, smoking during pregnancy, alcohol consumption during pregnancy, stress during pregnancy, and fetal sex. Abbreviations: GWG: gestational weight gain; OR: odds ratio; Q: quartile, SD: standard deviation.

### 3.4. Dietary Patterns and trajectories of Gestational Weight

Figure S2 shows the longitudinal relationship between the *a posteriori*-derived dietary patterns with trajectories of maternal weight during pregnancy, as the difference in weight (kg) between the 12.5% quantile (“quartile 1”) and the 37.5%, 62.5% and 87.5% quantiles (“quartiles” 2, 3 and 4, respectively) of adherence to the dietary pattern in normal weight women. Corresponding results for overweight women are displayed in Figure S3. In both normal weight and overweight women, most of the main effects of diet as well as the interaction terms with gestational age were not significant (Table S5). Only the “Margarine, sugar and snacks” pattern was significantly associated with higher weight in normal weight women (0.30 (95% CI 0.07; 0.52)) throughout pregnancy and the “Nuts, high-fiber cereals and soy” pattern was associated with slightly slower weight gain in normal weight women (−0.01 (95% CI −0.02; −0.00)).

### 3.5. Sensitivity Analyses

The results of the sensitivity analyses are presented in Table S3 for normal weight women and in Table S4 for overweight women. Additional adjustment for energy intake resulted in little attenuation of the effect estimate of the “Vegetable, oil and fish” pattern with GWG until early-third trimester, however the association remained statistically significant. Further adjustment of estimated fetal weight did not change the effect estimates of any dietary pattern with GWG in normal weight or overweight women. The results did not alter greatly after exclusion of women who vomited more than once per week, or exclusion of women with pre-existing comorbidities or pregnancy complications. The evaluation of maximum GWG showed that normal weight women with high adherence to the “Vegetable, oil and fish” pattern had 29 g/week (95% CI 2; 57) higher maximal GWG than women with low adherence. In addition, normal weight women in the highest quartile of the “Dutch Healthy Diet Index” had a 28 g/week (95% CI −55; −1) lower maximal GWG than women in quartile 1. The association between dietary patterns and GWG was not modified by educational level. For household income, women with higher household income (≥2200 Euro/month) and higher scores on the “Dutch Healthy Diet Index” pattern had lower GWG (*p*-value = 0.01 per 1SD score) than women with higher income and lower scores on this dietary pattern, whereas no association was found in women with lower household income (*p*-value = 0.11).

Evaluating adequacy of GWG using weekly GWG instead of total GWG resulted in a higher percentage women being classified as having “excessive GWG” (57% *vs.* 43%). However, also with this different definition a high adherence to the “Margarine, sugar and snacks” pattern was associated with a higher prevalence excessive weekly GWG. Nonetheless, high adherence to this pattern was also associated with higher prevalence of inadequate weekly GWG, although without dose-response association (Table S6).

Six years after childbirth, women had gained on average 3.4 kg (IQR: 0.4; 7.0) compared to their pre-pregnancy weight (*n* = 2247). The median (IQR) long-term weight gain was significantly different between the categories of GWG adequacy: women with inadequate GWG gained 2.2 kg (−0.6; 5.2), those with adequate GWG gained on average 2.6 kg (0.2; 5.2), and women with excessive GWG were 4.6 kg (1.4; 8.8) heavier (*F*-test 27.5, *p*-value < 0.001). The weight 6 years after childbirth was highly correlated with the weight at *the third visit* in pregnancy (*R* = 0.85; *p*-value < 0.001).

## 4. Discussion

### 4.1. Summary of Main Findings

Our results from a population-based Dutch cohort suggest that specific *a posteriori*-derived dietary patterns have a limited influence in early-pregnancy GWG, the prevalence of excessive GWG, and weight development in pregnancy. We found neither consistent associations of any dietary pattern with the prevalence of inadequate GWG, nor was the *a priori*-defined dietary pattern associated with GWG.

### 4.2. Interpretation and Comparison with Other Studies

The association of dietary patterns during pregnancy with GWG has been evaluated previously in a few studies [15,16,17,18], but these studies did not evaluate longitudinal development of gestational weight and were conducted in different populations. Uusitalo *et al.*, found that higher adherence to an *a posteriori*-derived dietary pattern characterized by high intake of sweets, fast food and snacks was associated with higher weekly GWG [15]. In line with these results [15], we found that higher adherence to the unhealthy “Margarine, sugar and snacks” pattern was associated with higher prevalence of excessive GWG. Additionally, Uusitalo *et al.*, reported that a pattern that was high in vegetables, fish and fruits was not associated with GWG [15]. In contrast, we found that the “Vegetable, oil and fish” pattern, a relatively healthy pattern, was associated with higher GWG, particularly in early pregnancy.

In our study, the *a priori*-defined “Dutch Healthy Diet Index” pattern was not consistently associated with any measure of GWG. This result was in accordance with two studies showing no relationship between the *a priori*-defined “US healthy eating index of 2005” (HEI-2005) and the “Alternate Healthy Eating Index, slightly modified for pregnancy” (AHEI-P) with the prevalence of inadequate or excessive GWG [16,17]. Nevertheless, a large population-based cohort study of over 66,000 participants found that high adherence to the *a priori*-defined “New Nordic Diet score” was associated with a 7% lower prevalence of excessive GWG in normal weight women, compared with low adherence [18]. The inconsistent significant associations between the *a priori*-defined dietary patterns may be due to different items that were included in the diet scores, whereas the “New Nordic Diet score” contained items on meal patterns and the type of beverages consumed, among others [18]; these items were not evaluated in our Dutch Healthy Diet Index, nor in other *a priori*-defined dietary patterns [16,17].

The association of *a posteriori*-derived dietary patterns with weight trajectories over pregnancy has not been evaluated previously, to our knowledge. Studying this association longitudinally has the advantage that all available weight measurements can be used, and takes into account the correlation between these measurements. In addition, weekly GWG is not constant over pregnancy and differs considerably by individual [28,36], which complicates cross-sectional comparisons of GWG. Our longitudinal analysis showed that women with higher adherence to the “Nuts, high-fiber and soy” pattern had a more moderate increase in weight during pregnancy than did women with low adherence to this dietary pattern, although absolute differences were small.

Results from both observational and interventional studies indicated that women with higher energy intake had higher GWG compared with women who have lower energy intake [8], results that were also found in our cohort [4]. In our analyses, the association of the “Vegetable, oil and fish” pattern remained significantly associated with GWG after additional adjustment for energy intake. This may indicate that dietary patterns are associated with GWG beyond energy intake.

Evaluating weight gain in pregnancy is important because GWG has been associated with many adverse pregnancy and birth outcomes. Gaining excessive weight during pregnancy can have short-term consequences such as delivery complications, and giving birth to a child that is large for its gestational age [3,4,28]. Additionally, it has been associated with long-term health consequences including post-partum obesity of the mother [37] due to retaining their excess fat mass, and childhood obesity [4]. Indeed, in our population, six years after childbirth women had gained on average 3.4 kg from their pre-pregnancy weight.

Weight gain during pregnancy consists of several maternal and fetal components that contribute differently to GWG over time [12]. For example, during the first half of pregnancy, maternal fat gain is a major contributor of GWG [38,39], and most of the fat gain that takes place during pregnancy is in that period [40]. In our study, the higher GWG in women with high adherence to the “Vegetable, oil and fish” pattern could not be explained by fetal growth and was mainly found in early pregnancy, meaning this higher GWG is likely due to maternal components, e.g., fat mass.

Our results for normal weight women differed from those for overweight women, particularly for the “Vegetable, oil and fish” pattern and for the “Nuts, high-fiber cereals and soy” pattern. Similarly, Hillesund *et al.* reported differential associations for women below and above a BMI of 25 kg/m^2^ [18]. These differential findings may be explained by different reporting of dietary intake [41] or by differing contribution of the individual components of GWG for normal weight and overweight women [42]. In addition, our longitudinal analyses showed that over the whole course of pregnancy, normal weight women with higher adherence to the “Margarine, sugar and snacks” pattern tend to be heavier than women with lower adherence.

### 4.3. Strengths and Limitations

A strength of our study is that we used a comprehensive approach to analyze the relation between diet and GWG by evaluating the associations of dietary patterns with (1) GWG during different phases in pregnancy, (2) adequacy of GWG, and (3) trajectories of gestational weight. Another strength is the use of two distinct methods to define dietary patterns, which enabled us to evaluate the effects of dietary patterns derived by a data-driven and by a hypothesis-driven approach. Dietary patterns represent the combined effects of all foods consumed [13], which may lead to a more powerful effect than the effects of the individual components, although it may also have led to a dilution of the effects of individual components that are associated with GWG [43]. For example, the food groups of vegetables and high-fat dairy products were strongly associated with the “Vegetable, oil and fish” dietary pattern. Yet, higher intake of fruits and vegetables has been associated with lower GWG [44], whereas dairy products were associated with higher GWG [9,10]. Consequently, this may result in an overall null effect of the dietary pattern. Furthermore, imputing the missing covariate values in the Bayesian framework allowed us to use all available information in the imputation. Especially in settings with a longitudinal outcome, imputation methods that are available in standard software and, hence, are more commonly used, often fail to appropriately include the outcome into the imputation procedure which may lead to severely biased results [31]. Other strengths of our study are its population-based design, the collection of numerous covariates, and that the population was restricted to women of Dutch ancestry. We excluded women with other ethnicities to minimize measurement error, since the FFQ was designed to evaluate a Dutch diet. However, this restriction may have reduced the generalizability of our results to other ethnicities.

Our study also has some limitations. First, maternal weight before pregnancy as well as maximum weight were obtained using questionnaires, which may have resulted in a larger measurement error. Although we found no indication of systematic measurement error, random error may have resulted in loss of precision in GWG assessment. Furthermore, we were not able to calculate GWG per trimester because we did not have weight measurements at the required time points and the available data was insufficient for imputing those values. Another limitation is the lack of information on the separate components of GWG, in particular maternal fat mass, and the lack of information on postpartum maternal weight. Future studies should collect detailed information on maternal body composition during pregnancy or measure the participants’ weight a few weeks postpartum to evaluate associations with the different components of GWG. Also, we could not use information on absolute dietary intake because dietary information collected using an FFQ does not provide this information. However, FFQs have been shown to be accurate in ranking participants according to their dietary intake [45]. Furthermore, we assessed maternal diet only once during pregnancy and were therefore not able to account for changes in dietary intake. Nevertheless, dietary patterns and macronutrient composition may not change largely during pregnancy despite an increased energy intake [46,47]. Additionally, we found that our results did not change after excluding women who may have altered their dietary intake due to illness or vomiting. Finally, the numerous statistical analyses performed may have resulted in chance findings (*type I error*). However, our results for weight trajectories and early-pregnancy GWG remained statistically significant when a more stringent alpha-level was used (alpha-level 0.05/4 = 0.0125).

### 4.4. Conclusions and Implications

In conclusion, our results suggest that dietary composition during pregnancy may play a role in GWG in early pregnancy but has limited influence on total GWG in a population of Dutch women. The strength of the associations between dietary patterns and GWG differs for different definitions of dietary patterns and GWG. This suggests that the relationship between dietary patterns and GWG may be complex and may need further elucidation in order to facilitate the development of dietary guidelines during pregnancy and to adequately advise pregnant women on their diet.

## Supplementary Materials

The following are available online at www.mdpi.com/2072-6643/7/11/5476/s1, Figure S1: Bland Altman plots for self-reported pre-pregnancy weight and maximum weight in pregnancy. (A) Bland-Altman plot for pre-pregnancy weight and weight during the *first visit* (*n* = 2425); (B) Bland-Altman plot for maximum weight in pregnancy and weight during the *third visit* (*n* = 2177), Figure S2: Trajectories of gestational weight in normal weight women (*n* = 2564). The figure shows the development of weight during pregnancy in normal weight women as estimated by the linear mixed model. Trends are plotted for 37.5%, 62.5%, and 87.5% quantiles (denoted as quartiles 2 until 4) as compared with the 12.5% quantile (denoted quartile 1). Adjusted for gestational age at measurements, age, educational level, household income, parity, smoking during pregnancy, alcohol consumption during pregnancy, stress during pregnancy, and fetal sex. Panel A displays effect estimates with 95% CI. Panel B zooms in on the effect estimates, Figure S3: Trajectories of gestational weight in overweight women (*n* = 810). The figure shows the development of weight during pregnancy in overweight women as estimated by the linear mixed model. Trends are plotted for 37.5%, 62.5%, and 87.5% quantiles (denoted as quartiles 2 until 4) as compared with the 12.5% quantile (denoted quartile 1). Adjusted for gestational age at measurements, age, educational level, household income, parity, smoking during pregnancy, alcohol consumption during pregnancy, stress during pregnancy, and fetal sex. Panel A displays effect estimates with 95% CI. Panel B zooms in on the effect estimates, Table S1: Food groups and its food components, Table S2: Modified version of the Dutch Healthy Diet-index, Table S3: Sensitivity analyses in normal weight women (*n* = 2141), Table S4: Sensitivity analyses in overweight women (*n* = 674), Table S5: Gestational weight trajectories in normal weight and overweight women (*n* = 3374), Table S6: Association of dietary patterns with adequacy of weekly gestational weight gain (*n* = 2745).
