# Supplementary material for: A Priori and a Posteriori Dietary Patterns during Pregnancy and Gestational Weight Gain: The Generation R Study
Source: Nutrients. 2015 Nov 12;7(11):9383–99. doi: 10.3390/nu7115476 (PMC4663604; doi:10.3390/nu7115476)
Supplement: Supplementary file 1 [file nutrients-07-05476-s001.docx]

**Supplementary Material: A Priori and a Posteriori Dietary Patterns during Pregnancy and Gestational Weight Gain: the Generation R Study**

Myrte J. Tielemans ^1,2,^*, Nicole S. Erler ^1,3^, Elisabeth T.M. Leermakers ^1,2^, Marion van den Broek ^1^, Vincent W.V. Jaddoe ^1,2,4^, Eric A.P. Steegers ^5^, Jessica C. Kiefte-de Jong ^1,6^ and Oscar H. Franco ^1^

|  |
| --- |
| (A) |
|  |
| (B) |

**Figure S1.** Bland Altman plots for self-reported pre-pregnancy weight and maximum weight in pregnancy. (**A**) Bland-Altman plot for pre-pregnancy weight and weight during *the first visit* (*n* = 2425); (**B**) Bland-Altman plot for maximum weight in pregnancy and weight during *the third visit* (*n* = 2177).


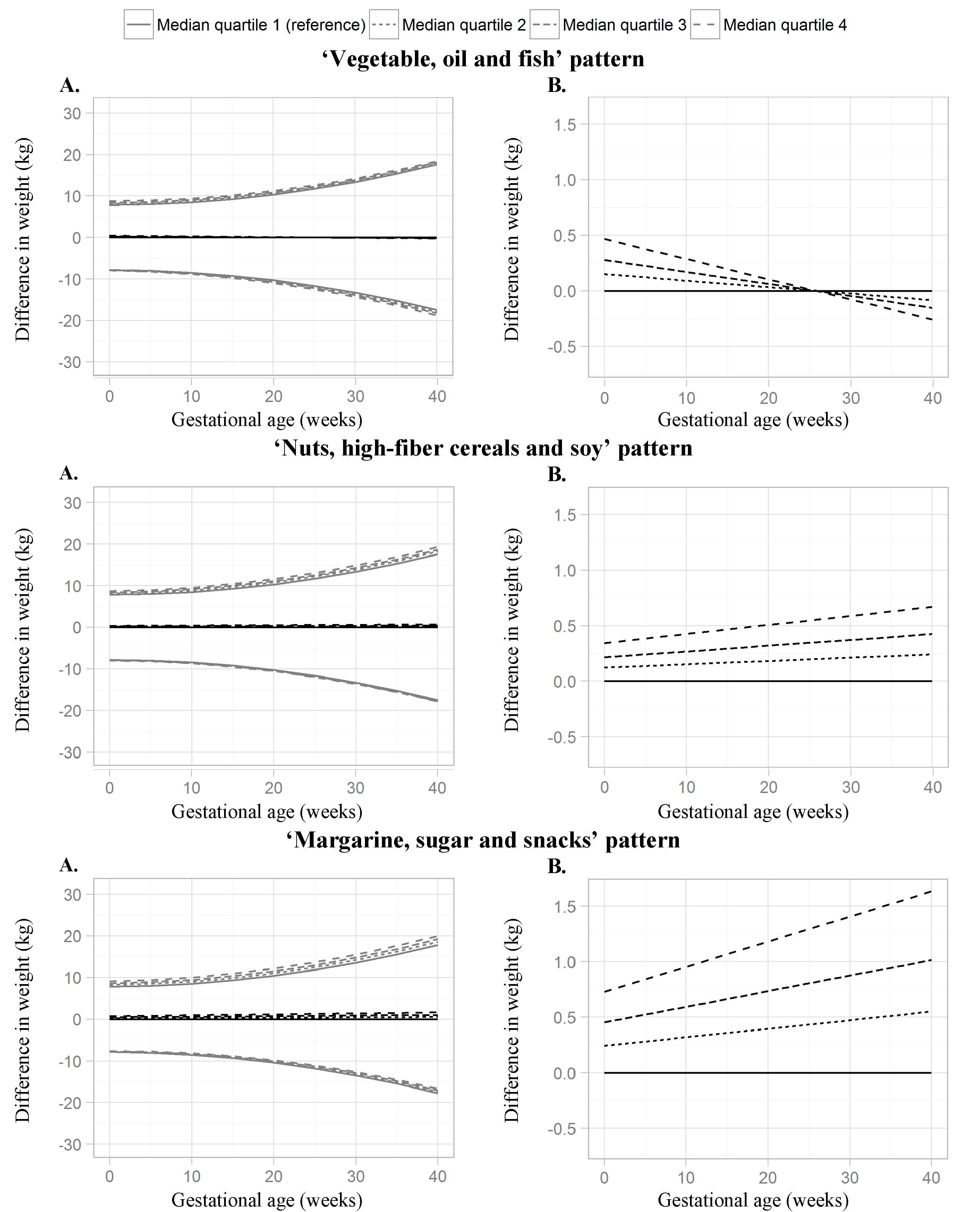


**Figure S2.** Trajectories of gestational weight in normal weight women (*n* = 2564). The figure shows the development of weight during pregnancy in normal weight women as estimated by the linear mixed model. Trends are plotted for 37.5%, 62.5%, and 87.5% quantiles (denoted as quartiles 2 until 4) as compared with the 12.5% quantile (denoted quartile 1). Adjusted for gestational age at measurements, age, educational level, household income, parity, smoking during pregnancy, alcohol consumption during pregnancy, stress during pregnancy, and fetal sex. Panel A displays effect estimates with 95% CI. Panel B zooms in on the effect estimates.


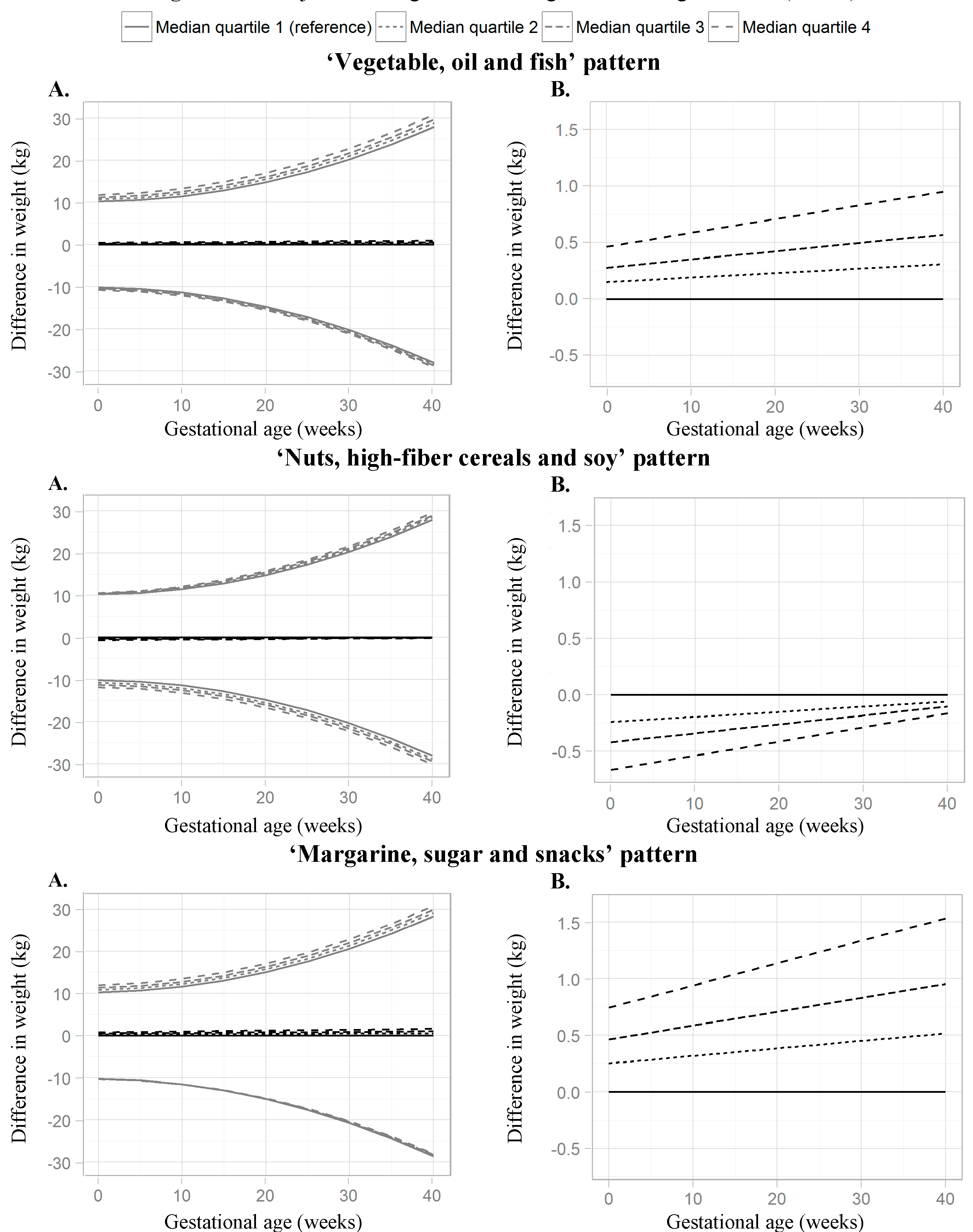


**Figure S3.** Trajectories of gestational weight in overweight women (*n* = 810). The figure shows the development of weight during pregnancy in overweight women as estimated by the linear mixed model. Trends are plotted for 37.5%, 62.5%, and 87.5% quantiles (denoted as quartiles 2 until 4) as compared with the 12.5% quantile (denoted quartile 1). Adjusted for gestational age at measurements, age, educational level, household income, parity, smoking during pregnancy, alcohol consumption during pregnancy, stress during pregnancy, and fetal sex. Panel A displays effect estimates with 95% CI. Panel B zooms in on the effect estimates.

**Table S1.** Food groups and its food components ^1^.

| **Food Group** | **Food Components** |
| --- | --- |
| Potatoes and other tubers | Potatoes (cooked or fried) and french fries |
| Vegetables | Endive, purslane, turnip tops, lettuce, chard, spinach, chicory, eggplant, avocado, cucumber, paprika, corn, pickle, tomatoes, beans, snow peas, courgette, tomato sauce, carrots, beetroots, cabbages, mushrooms, green peas, onions, leek, garlic, bean sprouts, celery, atjar tjampoer and crudités |
| Fruits | Apple, strawberries, apricot, pineapple, red berries, banana, blackberries, lemon, grapes, raspberries, cherries, grapefruit, tangerine, peach, pear, prunes, orange, applesauce, kiwi, mango, rhubarb, melon, nectarine, canned fruit and dried fruit |
| Dairy products—high fat | Whole milk, full-fat yoghurt drink, full-fat yogurt,  cheese 40–60+, cream cheese, full-fat quark, pudding,  sour cream, whipped cream and crème fraiche |
| Dairy products—low fat | Skimmed and semi-skimmed milk, skimmed and  semi-skimmed yoghurt drink, skimmed and  semi-skimmed yogurt, cheese 20–30+ and low-fat quark |
| Cereals—high fiber | Brown rice, whole wheat pasta, whole wheat/brown/rye  bread, muesli, seitan, oatmeal, wheat germ, and bran |
| Cereals—low fiber | White rice, couscous, bulgur, white bread, rusk, croissants,  corn flakes, crackers, rice cake, toast, pancakes,  raisin bread, bami and white pasta |
| Meat and meat products | Red meat, poultry, organ meat and meat products |
| Fish and shellfish | Fish, fish products and shellfish |
| Eggs and egg products | Egg (cooked or fried) |
| Vegetable oils | Olive oil, nut oil, salad oil, sesame oil,  sunflower oil, soybean oil and peanut oil |
| Margarine and butter | Margarine (solid and liquid) and butter |
| Sugar and confectionary and cakes | Chocolate, candy, chocolate sprinkles, cake, pastry and biscuits |
| Snacks | Peanuts, beer nuts, trail mix, pretzels and chips |
| Coffee and tea | Coffee, cappuccino, espresso, English tea, green tea and herbal tea |
| Sugar-containing beverages | Coke/Fanta/Sprite soft drinks, isotonic  drinks, fruit juices and vegetable juices |
| Light soft drinks | Coke/Fanta/Sprite light soft drinks and water |
| Alcoholic beverages | Beer, wine, mixed drinks and liquor |
| Condiments and sauces | Chili/tomato/barbecue/shaslick/peanut/garlic/whisky/soy/salad sauce, salad dressing, mayonnaise, fish/egg/meat/Russian salad, sandwich spread, marmite, salt, spices/herbs and flavour powder |
| Soups and bouillon | Soup or bouillon with or without meat, meal soup and lentil soup |
| Nuts, seeds and olives | Nut butter, tahini, poppy seed, sesame, pumpkin seeds,  sunflower seeds, pine nuts, mixed seeds and olives |
| Soy products | Tofu, tempeh, soymilk, soy chunks, soy  dessert, vegetable burgers and quorn |
| Legumes | Legumes and tempeh |

^1^ Reprinted with permission from van den Broek *et al.*, [1]. The food groups were primarily created based on the Dutch National Food Consumption Survey.

**Table S2.** Modified version of the Dutch Healthy Diet-index ^1^.

| **Components of the Dutch Healthy Diet-Index** | | **Minimum Score (0)** | **Maximum Score (10)** |
| --- | --- | --- | --- |
| 1 | Physical activity (daily) | NA | NA |
| 2 | Vegetable (g/day) | 0 | ≥200 |
| 3 | Fruit (g/day) ^a^ | 0 | ≥200 |
| 4 | Fiber (g/4.2MJ/day) | 0 | ≥14 |
| 5 | Fish (g/day) | 0 | 34.3 ^b^ |
| 6 | Saturated fat (E%) | ≥16.6 | <10 |
| 7 | Trans fatty acid (E%) | NA | NA |
| 8 | Acidic drinks and foods (servings/day) | NA | NA |
| 9 | Sodium (mg/day) | ≥2450 | <1680 |
| 10 | Alcohol (g/day) | NI | NI |

^1^ Adapted from van Lee *et al.*, [2]; ^a^ Fruit without fruit juices; ^b^ Based on two portions of 120 g fish per week [3]; Abbreviations: NA: not available; NI: not included.

**Table S3.** Sensitivity analyses in normal weight women (*n* = 2141) ^1^.

| **Dietary Pattern Quartile** | **Gestational Weight Gain until Early Third Trimester of Pregnancy** | **1. Additionally Adjusted for Energy Intake ^2^** | **2. Additional Adjustment for Estimated Fetal Weight** | **3. Excluding Women  with Comorbidities or Pregnancy Complications ^3^** | **4. Excluding Women Vomiting > 1 Times per Week in Previous Three Months** | **Maximal Gestational  Weight Gain ^4^** |
| --- | --- | --- | --- | --- | --- | --- |
|  | ***n* = 2141** | ***n* = 2141** | ***n* = 2120** | ***n* = 1937** | ***n* = 1890** | ***n* = 1492** |
|  | **“Vegetable, Oil and Fish” Pattern** | | | | | |
| Q1 (low) | *Reference* | *Reference* | *Reference* | *Reference* | *Reference* | *Reference* |
| Q2 | 16 (−0; 31) | 14 (−1; 30) | **16 (0; 32)** | **20 (3; 36)** | 13 (−4; 30) | 14 (−12; 41) |
| Q3 | 5 (−11; 21) | 2 (−15; 18) | 5 (−11; 21) | 7 (−10; 23) | 2 (−15; 19) | 4 (−23; 31) |
| Q4 (high) | **25 (9; 42) *** | **18 (0; 35)** | **25 (9; 41) *** | **27 (11; 44) *** | **20 (3; 37)** | **29 (2; 57)** |
| Per SD | ***p* = 0.02** | *p* = 0.29 | ***p* = 0.02** | ***p* < 0.01 *** | *p* = 0.11 | *p* = 0.19 |
|  | **“Nuts, High-Fiber Cereals and Soy” Pattern** | | | | | |
| Q1 (low) | *Reference* | *Reference* | *Reference* | *Reference* | *Reference* | *Reference* |
| Q2 | 10 (−6; 26) | 7 (−9; 23) | 11 (−5; 26) | 12 (−4; 29) | 11 (−6; 28) | 16 (−12; 44) |
| Q3 | −3 (−19; 14) | −6 (−23; 10) | 0 (−16; 16) | 0 (−17; 17) | −1 (−18; 16) | 25 (−3; 54) |
| Q4 (high) | −5 (−22; 11) | −12 (−29; 5) | −5 (−21; 11) | −2 (−19; 15) | −7 (−24; 10) | 10 (−19; 38) |
| Per SD | *p* = 0.51 | *p* = 0.14 | *p* = 0.36 | *p* = 0.66 | *p* = 0.48 | *p* = 0.20 |

**Table S3.** *Cont.*

| **Dietary Pattern Quartile** | **Gestational Weight Gain until Early Third Trimester of Pregnancy** | **1. Additionally Adjusted for Energy Intake ^2^** | **2. Additional Adjustment for Estimated Fetal Weight** | **3. Excluding Women  with Comorbidities or Pregnancy Complications ^3^** | **4. Excluding Women Vomiting > 1 Times per Week in Previous Three Months** | **Maximal Gestational  Weight Gain ^4^** |
| --- | --- | --- | --- | --- | --- | --- |
|  | ***n* = 2141** | ***n* = 2141** | ***n* = 2120** | ***n* = 1937** | ***n* = 1890** | ***n* = 1492** |
|  | **“Margarine, Sugar and Snacks” Pattern** | | | | | |
| Q1 (low) | *Reference* | *Reference* | *Reference* | *Reference* | *Reference* | *Reference* |
| Q2 | 6 (−9; 21) | −1 (−19; 16) | 3 (−12; 18) | 6 (−10; 22) | 4 (−12; 20) | **27 (1; 53)** |
| Q3 | −0 (−16; 16) | −14 (−35; 7) | −2 (−17; 13) | 1 (−15; 17) | −3 (−20; 13) | 26 (−0; 53) |
| Q4 (high) | 11 (−5; 26) | −11 (−40; 17) | 9 (−7; 24) | 10 (−6; 26) | 7 (−9; 23) | 24 (−2; 50) |
| Per SD | *p* = 0.37 | *p* = 0.06 | *p* = 0.48 | *p* = 0.48 | *p* = 0.82 | *p* = 0.12 |
|  | **“Dutch Healthy Diet-Index” Pattern** | | | | | |
| Q1 (low) | *Reference* | *Reference* | *Reference* | *Reference* | *Reference* | *Reference* |
| Q2 | −9 (−24; 7) | −10 (−25; 6) | −8 (−23; 8) | −13 (−29; 3) | −12 (−28; 4) | −10 (−35; 16) |
| Q3 | −2 (−18; 13) | −3 (−19; 12) | −3 (−18; 13) | −4 (−20; 12) | −6 (−22; 10) | 4 (−22; 29) |
| Q4 (high) | −11 (−27; 5) | −11 (−27; 5) | −9 (−25; 6) | −16 (−33; 1) | −13 (−29; 4) | **−28 (−55; −1)** |
| Per SD | *p* = 0.13 | *p* = 0.13 | *p* = 0.19 | *p* =0.06 | *p* = 0.08 | *p* = 0.05 |

^1^ Values (regression coefficients with 95%-confidence interval) reflect the difference in gestational weight gain until early-third trimester (g/week) for quartile 2 until 4 relative to quartile 1 in normal weight women. *p*-Values correspond to the effect of 1SD increase in dietary pattern score. Adjusted for pre-pregnancy BMI, median gestational age at follow-up, age, educational level, household income, parity, smoking during pregnancy, alcohol consumption during pregnancy, stress during pregnancy, and fetal sex. ^2^ Further adjusted for energy intake (kcal/day). ^3^ Women excluded with pre-existing comorbidities (diabetes mellitus, hypercholesterolemia, hypertension, heart disease, thyroid disease, and systemic lupus erythematosus) and with pregnancy complications (pregnancy-induced hypertension, preeclampsia or gestational diabetes). ^4^ Gestational weight gain calculated from self-reported pre-pregnancy weight and maximum weight in pregnancy. Significant results are presented in bold (*p*-value < 0.05) and results with a *p*-value < 0.0125 with an asterisk (*).

**Table S4.** Sensitivity analyses in overweight women (*n* = 674) ^1^.

| **Dietary Pattern Quartile** | **Gestational Weight Gain until Early Third Trimester of Pregnancy** | **1. Additionally Adjusted for Energy Intake ^2^** | **2. Additional Adjustment for Estimated Fetal Weight** | **3. Excluding Women  with Comorbidities or Pregnancy Complications ^3^** | **4. Excluding Women Vomiting > 1 Times per Week in Previous Three Months** | **Maximal Gestational  Weight Gain ^4^** |
| --- | --- | --- | --- | --- | --- | --- |
|  | ***n* = 674** | ***n* = 674** | ***n* = 665** | ***n* = 532** | ***n* = 560** | ***n* = 425** |
|  | **“Vegetable, Oil and Fish” Pattern** | | | | |  |
| Q1 (low) | *Reference* | *Reference* | *Reference* | *Reference* | *Reference* | *Reference* |
| Q2 | 30 (−8; 68) | 29 (−8; 67) | 28 (−9; 65) | 27 (−13; 66) | 27 (−14; 68) | 11 (−40; 62) |
| Q3 | **40 (1; 79)** | 38 (−2; 77) | **41 (2; 80)** | 40 (−2; 81) | **44 (3; 86)** | 25 (−27; 77) |
| Q4 (high) | 26 (−15; 67) | 22 (−21; 65) | 25 (−15; 66) | 19 (−23; 61) | 38 (−6; 82) | 1 (−54; 55) |
| Per SD | *p* = 0.15 | *p* = 0.22 | *p* = 0.13 | *p* = 0.18 | *p* = 0.07 | *p* = 0.87 |
|  | **“Nuts, High-Fiber Cereals and Soy” Pattern** | | | | |  |
| Q1 (low) | *Reference* | *Reference* | *Reference* | *Reference* | *Reference* | *Reference* |
| Q2 | 1 (−38; 39) | 0 (−38; 39) | −0 (−38; 38) | −23 (−63; 17) | 6 (−38; 49) | 10 (−46; 67) |
| Q3 | −14 (−53; 25) | −14 (−54; 25) | −19 (−58; 19) | −8 (−50; 33) | −14 (−57; 29) | 47 (−9; 102) |
| Q4 (high) | −9 (−50; 32) | −10 (−53; 32) | −12 (−52; 28) | −12 (−55; 31) | −11 (−55; 33) | 15 (−41; 72) |
| Per SD | *p* = 0.49 | *p* = 0.45 | *p* = 0.38 | *p* = 0.48 | *p* = 0.38 | *p* = 0.70 |
|  | **“Margarine, Sugar and Snacks” Pattern** | | | | |  |
| Q1 (low) | *Reference* | *Reference* | *Reference* | *Reference* | *Reference* | *Reference* |
| Q2 | 11 (−26; 48) | 19 (−24; 61) | 10 (−27; 46) | 1 (−39; 40) | 7 (−33; 48) | −1 (−50; 48) |
| Q3 | 18 (−20; 55) | 31 (−21; 83) | 16 (−21; 53) | 26 (−12; 65) | 9 (−31; 49) | 8 (−42; 59) |
| Q4 (high) | 30 (−8; 68) | 51 (−17; 120) | 28 (−9; 66) | 38 (−2; 79) | 27 (−15; 69) | 22 (−31; 75) |
| Per SD | *p* = 0.36 | *p* =0.89 | *p* = 0.41 | *p* = 0.20 | *p* = 0.49 | *p* = 0.51 |

**Table S4.** *Cont.*

| **Dietary Pattern Quartile** | **Gestational Weight Gain until Early Third Trimester of Pregnancy** | **1. Additionally Adjusted for Energy Intake ^2^** | **2. Additional Adjustment for Estimated Fetal Weight** | **3. Excluding Women  with Comorbidities or Pregnancy Complications ^3^** | **4. Excluding Women Vomiting > 1 Times per Week in Previous Three Months** | **Maximal Gestational  Weight Gain ^4^** |
| --- | --- | --- | --- | --- | --- | --- |
|  | ***n* = 674** | ***n* = 674** | ***n* = 665** | ***n* = 532** | ***n* = 560** | ***n* = 425** |
|  | **“Dutch Healthy Diet-Index” Pattern** | | | | |  |
| Q1 (low) | *Reference* | *Reference* | *Reference* | *Reference* | *Reference* | *Reference* |
| Q2 | −3 (−40; 34) | −4 (−41; 34) | 1 (−36; 38) | −6 (−46; 34) | 8 (−32; 47) | −28 (−76; 21) |
| Q3 | 16 (−22; 54) | 15 (−22; 53) | 15 (−23; 52) | 11 (−29; 52) | 28 (−13; 68) | −5 (−55; 44) |
| Q4 (high) | 9 (−30; 48) | 9 (−30; 48) | 9 (−30; 48) | 11 (−30; 52) | −3 (−46; 40) | −9 (−62; 44) |
| Per SD | *p* = 0.84 | *p* = 0.83 | *p* = 0.82 | *p* = 0.89 | *p* = 0.50 | *p* = 0.84 |

^1^ Values (regression coefficients with 95%-confidence interval) reflect the difference in gestational weight gain until early third trimester (g/week) for quartile 2 until 4 relative to quartile 1 in overweight women. *p*-Values correspond to the effect of 1SD increase in dietary pattern score. Adjusted for pre-pregnancy BMI, median gestational age at follow-up, age, educational level, household income, parity, smoking during pregnancy, alcohol consumption during pregnancy, stress during pregnancy, and fetal sex. ^2^ Further adjusted for energy intake (kcal/day). ^3^ Women excluded with pre-existing comorbidities (diabetes mellitus, hypercholesterolemia, hypertension, heart disease, thyroid disease, and systemic lupus erythematosus) and with pregnancy complications (pregnancy-induced hypertension, preeclampsia or gestational diabetes). ^4^ Gestational weight gain calculated from self-reported pre-pregnancy weight and maximum weight in pregnancy. Significant results are presented in bold (*p*-value < 0.05) and results with a *p*-value < 0.0125 with an asterisk (*).

**Table S5.** Gestational weight trajectories in normal weight and overweight women (*n* = 3374) ^1^.

| **Normal Weight Women (*n* = 2564)** | **Mean** | **95%CI** |
| --- | --- | --- |
| “Vegetable, oil and fish” pattern | 0.217 | −0.010; 0.443 |
| “Nuts, high-fiber cereals and soy” pattern | 0.151 | −0.084; 0.395 |
| “Margarine, sugar and snacks” pattern | **0.300** | **0.074; 0.520 *** |
| Gestational age (at measurement in weeks) | **0.131** | **0.121; 0.142 *** |
| Gestational age × gestational age | **0.008** | **0.007; 0.008 *** |
| “Vegetable, oil and fish” pattern × gestational age | -0.001 | −0.007; 0.005 |
| “Nuts, high-fiber cereals and soy” pattern × gestational age | **−0.010** | **−0.016; −0.004 *** |
| “Margarine, sugar and snacks” pattern × gestational age | 0.006 | −0.000; 0.012 |
| **Overweight women (*n* = 810)** | **Mean** | **95%CI** |
| “Vegetable, oil and fish” pattern | 0.263 | −0.180; 0.717 |
| “Nuts, high-fiber cereals and soy” pattern | −0.306 | −0.767; 0.140 |
| “Margarine, sugar and snacks” pattern | 0.319 | −0.074; 0.714 |
| Gestational age (at measurement in weeks) | **0.099** | **0.081; 0.118 *** |
| Gestational age × gestational age | **0.008** | **0.007; 0.008 *** |
| “Vegetable, oil and fish” pattern × gestational age | 0.005 | −0.006; 0.016 |
| “Nuts, high-fiber cereals and soy” pattern × gestational age | 0.007 | −0.004; 0.018 |
| “Margarine, sugar and snacks” pattern × gestational age | 0.005 | −0.005; 0.015 |

^1^ The table shows the posterior mean and 95% credible intervals (95%CI) of the longitudinal analysis of *a posteriori*-derived dietary patterns and weight development in pregnancy in normal weight and overweight women using Bayesian linear mixed models. The model included the following variables: the *a posteriori*-derived dietary patterns, gestational age at measurement, gestational age at measurement × gestational age at measurement, interaction terms of the dietary patterns with gestational age at measurement, age, educational level, household income, parity, smoking during pregnancy, alcohol consumption during pregnancy, stress during pregnancy, and fetal sex. Significant results are presented in bold (*p*-value < 0.05) and results with a *p*-value <0.0125 with an asterisk (*).

**Table S6.** Association of dietary patterns with adequacy of weekly gestational weight gain (*n* = 2745) ^1^.

| **Dietary Pattern Quartile** | **Inadequate Weekly GWG (*n* = 437)** | **Adequate Weekly GWG (*n* = 753)** | **Excessive Weekly GWG (*n* = 1555)** |
| --- | --- | --- | --- |
|  | **OR (95%CI)** |  | **OR (95%CI)** |
|  | **“Vegetable, Oil and Fish” Pattern** | | |
| Q1 (low) | *Reference* | *Reference* | *Reference* |
| Q2 | 0.81 (0.57; 1.16) | *Reference* | 0.99 (0.76; 1.29) |
| Q3 | 0.83 (0.58; 1.18) | *Reference* | 0.89 (0.68; 1.16) |
| Q4 (high) | 0.97 (0.67; 1.41) | *Reference* | 0.85 (0.64; 1.13) |
| Per SD | *p* = 0.92 |  | *p* = 0.11 |
|  | **“Nuts, High-Fiber Cereals and Soy” Pattern** | | |
| Q1 (low) | *Reference* | *Reference* | *Reference* |
| Q2 | 1.12 (0.79; 1.59) | *Reference* | 1.16 (0.89; 1.51) |
| Q3 | 0.86 (0.59; 1.24) | *Reference* | 0.99 (0.76; 1.30) |
| Q4 (high) | 1.10 (0.76; 1.61) | *Reference* | 1.16 (0.87; 1.55) |
| Per SD | *p* = 0.83 |  | *p* = 0.20 |
|  | **“Margarine, Sugar And Snacks” Pattern** | | |
| Q1 (low) | *Reference* | *Reference* | *Reference* |
| Q2 | 1.17 (0.83; 1.65) | *Reference* | 1.13 (0.88; 1.47) |
| Q3 | 0.92 (0.65; 1.32) | *Reference* | 1.10 (0.85; 1.43) |
| Q4 (high) | **1.49 (1.05; 2.11)** | *Reference* | **1.32 (1.01; 1.72)** |
| Per SD | *p* = 0.19 |  | *p* = 0.29 |

**Table S6.** *Cont.*

| **Dietary Pattern Quartile** | **Inadequate Weekly GWG (*n* = 437)** | **Adequate Weekly GWG (*n* = 753)** | **Excessive Weekly GWG (*n* = 1555)** |
| --- | --- | --- | --- |
|  | **“Dutch Healthy Diet-Index” Pattern** | | |
| Q1 (low) | *Reference* | *Reference* | *Reference* |
| Q2 | 0.79 (0.56;1.11) | *Reference* | 0.93 (0.72;1.20) |
| Q3 | 0.88 (0.62;1.25) | *Reference* | 1.10 (0.85;1.42) |
| Q4 (high) | 1.00 (0.69;1.43) | *Reference* | 1.02 (0.77;1.34) |
| Per SD | *p* = 0.84 |  | *p* = 0.63 |

^1^ Results from multivariable multinomial logistic regression analyses, based on imputed data. Low dietary pattern adherence (Q1) is the reference category for diet and adequate weekly GWG is the reference category for weekly GWG in the multinomial regression model. *p*-Values correspond to the effect of 1SD increase in dietary pattern score. Adjusted for pre-pregnancy BMI, gestational age at measurements, age, educational level, household income, parity, smoking during pregnancy, alcohol consumption during pregnancy, stress during pregnancy, and fetal sex. Significant results are presented in bold (*p*-value < 0.05) and results with a *p*-value <0.0125 with an asterisk (*). Abbreviations: CI: confidence interval; GWG: gestational weight gain; OR: odds ratio; SD: standard deviation; Q: quartile.

Reference

1. Van den Broek, M.; Leermakers, E.T.; Jaddoe, V.W.; Steegers, E.A.; Rivadeneira, F.; Raat, H.; Hofman, A.; Franco, O.H.; Kiefte-de Jong, J.C. Maternal dietary patterns during pregnancy and body composition of the child at age 6 y: The generation R study. *Am. J. Clin. Nutr.* **2015**, *102*, 873–880.
2. Van Lee, L.; Geelen, A.; van Huysduynen, E.J.; de Vries, J.H.; van’t Veer, P.; Feskens, E.J. The Dutch healthy diet index (DHD-index): An instrument to measure adherence to the Dutch guidelines for a healthy diet. *Nutr. J.* **2012**, *11*, doi:10.1186/1475-2891-11-49.
3. Donders-Engelen, M.; van der Heijden, L. *Maten, Gewichten en Codenummers 2003 (Measures, Weights and Code Numbers 2003)*; Wageningen UR, Vakgroep Humane Voeding Wageningen and TNO Voeding: Zeist, the Netherlands, 2003.
